# Pollen and Phytoliths from Fired Ancient Potsherds as Potential Indicators for Deciphering Past Vegetation and Climate in Turpan, Xinjiang, NW China

**DOI:** 10.1371/journal.pone.0039780

**Published:** 2012-06-25

**Authors:** Yi-Feng Yao, Xiao Li, Hong-En Jiang, David K. Ferguson, Francis Hueber, Ruby Ghosh, Subir Bera, Cheng-Sen Li

**Affiliations:** 1 State Key Laboratory of Systematic and Evolutionary Botany, Institute of Botany, Chinese Academy of Sciences, Beijing, China; 2 School of Guoxue, Renmin University of China, Beijing, China; 3 Department of Scientific History and Archaeometry, Graduate University of Chinese Academy of Sciences, Beijing, China; 4 Department of Paleontology, University of Vienna, Vienna, Austria; 5 Department of Paleobiology, National Museum of Natural History, United States National Museum, Washington DC, United States of America; 6 Birbal Sahni Institute of Palaeobotany, Lucknow, India; 7 Department of Botany, University of Calcutta, Kolkata, India; The Pennsylvania State University, United States of America

## Abstract

It is demonstrated that palynomorphs can occur in fired ancient potsherds when the firing temperature was under 350°C. Pollen and phytoliths recovered from incompletely fired and fully fired potsherds (ca. 2700 yrs BP) from the Yanghai Tombs, Turpan, Xinjiang, NW China can be used as potential indicators for reconstructing past vegetation and corresponding climate in the area. The results show a higher rate of recovery of pollen and phytoliths from incompletely fired potsherds than from fully fired ones. Charred phytoliths recovered from both fully fired and incompletely fired potsherds prove that degree and condition of firing result in a permanent change in phytolith color. The palynological data, together with previous data of macrobotanical remains from the Yanghai Tombs, suggest that temperate vegetation and arid climatic conditions dominated in the area ca. 2700 yrs BP.

## Introduction

Conventional analyses of pollen and phytoliths from the sub-surface sediments of archaeological sites are increasingly gaining importance in exploring ancient vegetation and environments [1]–[5]. In addition, analyses of potsherds may prove to be another useful proxy in this regard. The potters mostly utilized clay for making earthenware from a suitable source (e.g., river bank, pond or lake) around their habitation, and palynomorphs in the surface sediments used for pottery making may reflect the vegetation growing around the habitation site. Palynomorphs are usually destroyed during the firing process, but poorly fired or incompletely fired potsherds are occasionally found, which may yield pollen and non-pollen palynomorphs [6].

In recent times, there have been reports of palynomorphs recovered from incompletely fired potsherds and terracotta figurines. Ghosh *et al.* (2006) studied palynomorphs from incompletely fired ancient potsherds from an excavation site (ca. 3300–2000 yrs BP) in Gangetic West Bengal, India, and inferred that a tropical, moist deciduous forest existed in the surrounding area at that time [6]. Based on comparative palynological studies of fragments from terracotta warrior and horse figures and soil samples from the Qin Dynasty layer of the Qin Shihuang Mausoleum, Hu *et al.* (2007) revealed that the terracotta horses were produced at a locality near the mausoleum, whereas the warriors came from a site that was further afield [7].

In addition to the study of palynomorphs, phytoliths (i.e., biogenic siliceous bodies) play a significant role in the reconstruction of past vegetation and environment at a given site [1], [8]–[11]. Study of these tiny siliceous particles is of immense importance for tracing the ancient vegetation, climate, and evolution of agriculture, especially when recovery of spores and pollen grains is poor [1]. In addition, *in situ* macrobotanical remains (e.g., seeds, fruits, charcoal, wood) from archaeological sites are also widely used to elucidate such factors as the ancient vegetation, environment, human-plant relations, human diet, plant domestication, and cultural contacts [1].

In the present study, pollen and phytoliths extracted from ancient potsherds (ca. 2700 yrs BP) found in the archaeological site of the Yanghai Tombs, Turpan, Xinjiang, NW China are used as potential indicators not only of the ancient vegetation and climate of the area but also to establish a relationship between the rate of recovery of pollen and phytoliths with the degree and duration of the firing process for the ancient pots.

## Materials and Methods

### Ethics statement

All necessary permits were obtained for the described field studies and were granted by the Academia Turfanica of Xinjiang Uygur Autonomous Region.

### Research site

**Figure 1:**
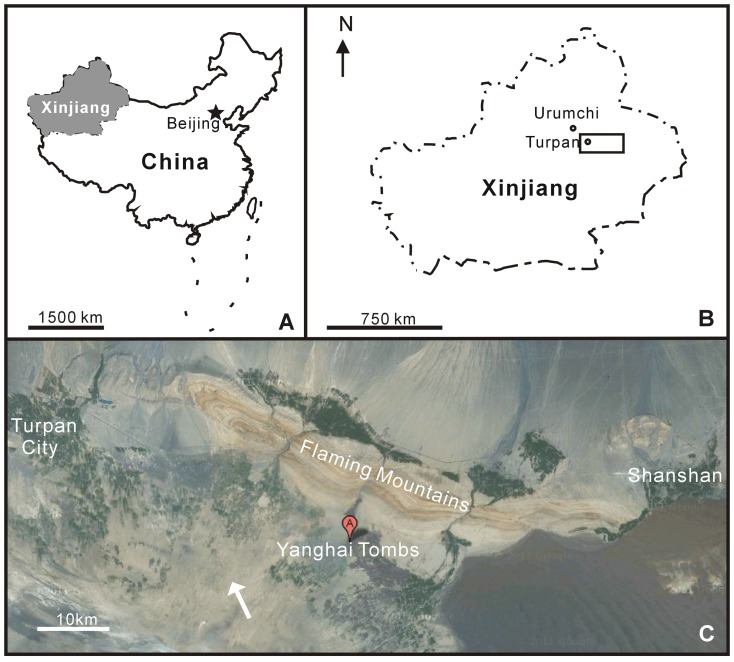
Maps showing the location of study site. (A) the location of Xinjiang in China, (B) the location of Turpan (the rectangular area) (revised from [43]), and (C) the location of the Yanghai Tombs in Xinjiang (from http://maps. google.com, white arrow represents the Gobi Desert).

The studied materials were collected from the Yanghai Tombs, Turpan, which are situated in eastern Xinjiang, NW China, approximately 180 km southeast of Urumqi (Fig. 1). Turpan lies in an intermontane basin surrounded by Bogeda Mountains (average elevation above 3500 m) to the north, Kalawucheng Mountains (3500–4000 m) to the west, and Jueluotage Mountains (600–1500 m) to the south. The basin covers an area of approximately 50 000 km^2^, including a small oasis (7.8 km^2^) enclosed by the vast Gobi Desert (Fig. 1) [12] within the warm temperate zone with little rainfall throughout the year. The current mean annual temperature and precipitation are 13.3–13.9°C and 16.8 mm, respectively, while in the mountainous regions (above 3000 m), the annual rainfall can reach 400 mm [12].

Water for the oases and for agricultural irrigation is derived mainly from the melting snows of the Bogeda and Kalawucheng Mountains, including the abundant water supplied by the mountains through subterranean karez systems. The local people are dominantly Uygur, who mostly lead an agricultural and sedentary life in the oases [13]. They mainly plant cotton (*Gossypium hirsutum* L.), grape (*Vitis vinifera* L.), broomcorn (*Sorghum bicolor* (L.) Moench), watermelon (*Citrullus lanatus* (Thunb.) Matsumura et Nakai), sweet melon (*Cucumis melo* L.), Chinese jujube (*Ziziphus jujuba* Mill.), apricot (*Armeniaca vulgaris* Lam.), and pomegranate (*Punica granatum* L.). The local vegetation is characteristic of a saline meadow setting and is dominated by camel throne (*Alhagi pseudalhagi* (Bieb.) Desv.) along with a number of xeromorphic grasses and shrubs, including Chinese tamarisk (*Tamarix* sp.), Mongolian calligonum (*Calligonum mongolicum* Turcz.), caper (*Capparis spinosa* L.), thorny elaeagnus (*Elaeagnus* sp.), licorice (*Glycyrrhiza* sp.), saxaul (*Haloxylon ammodendron* (C.A. Meyer) Bunge), wolfberry (*Lycium ruthenicum* Murr.), foxtail-like sophora (*Sophora alopecuroides* L.), dogbane (*Apocynum venetum* L.), and Caspian Sea karelinia (*Karelinia caspica* (Pall.) Less.) [13], [14].

**Figure 2:**
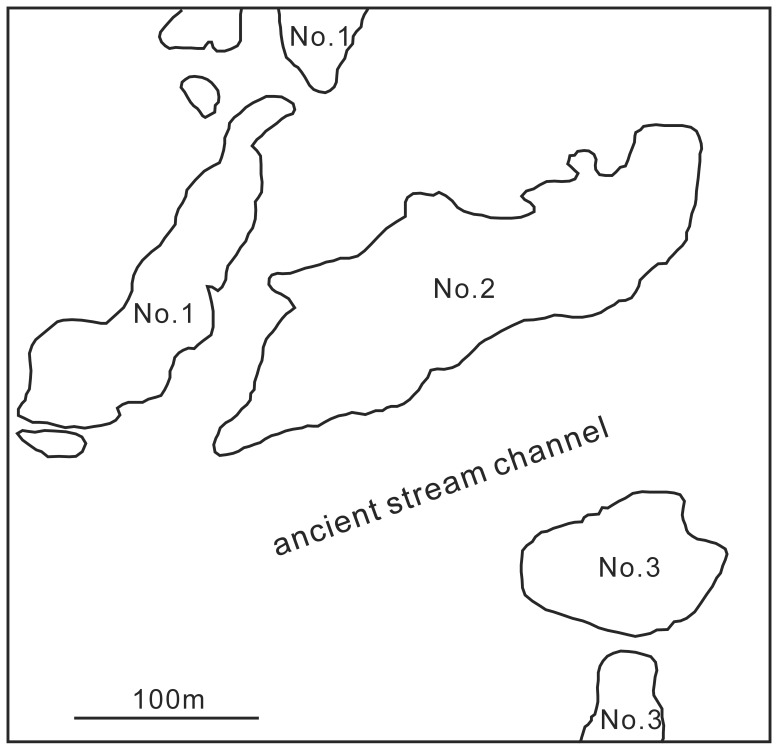
The location of the Group Nos. 1 to 3 in the Yanghai Tombs (revised from [**43**] **).**

Due to the arid climate, many mummies and funeral objects are well-preserved in the archaeological sites of this area [15]. The present archeological site, the Yanghai Tombs (42°48′–42°49′N, 89°39′–89°40′E), is located in the Turpan Basin at the foot of the Flaming Mountains (Fig. 1). The Tombs comprise three groups, Nos. 1–3 (Fig. 2), and belong to the Subeixi Culture, having an absolute age of between 3000 yrs BP and 2000 yrs BP. An initial radiocarbon date of 2500 yrs BP for plant materials collected from the tombs has been subsequently calibrated to 2700 yrs BP based on additional analyses of equestrian gear and correlation with tree ring data [16].

### Studied materials and methodology

**Figure 3:**
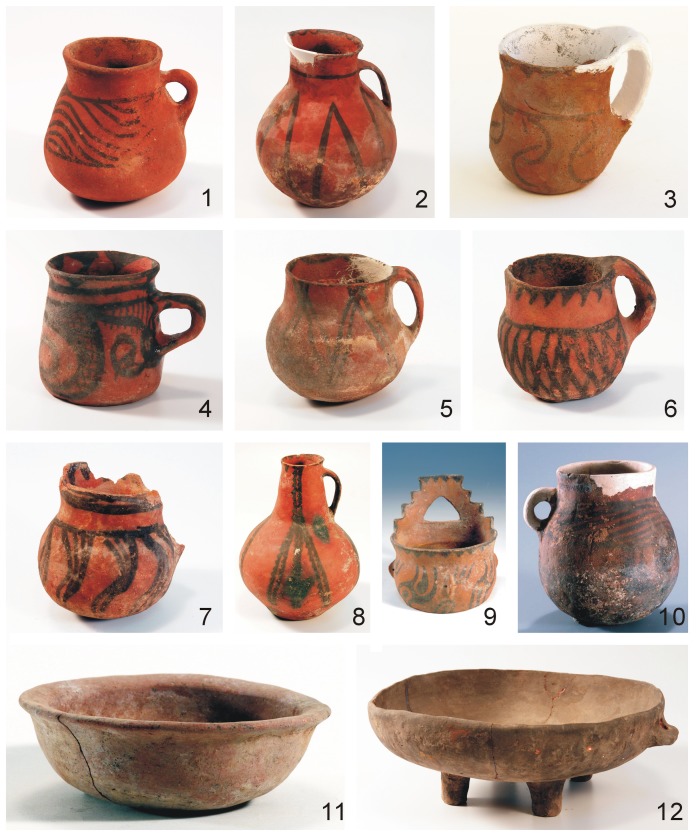
Painted pottery vessels excavated from the Yanghai Tombs (photographed by Yong-Bing Zhang). 1. Single-handled jar with waves (2003SAYIIM123.1), 2. Single-handled pot with folded lines (2003SAYIM195.1.), 3. Single-handled cup with arcuate sculpture (2003SAY II M221.1), 4. Single-handled cup with whirlpools (2003SAY IIM205.5), 5. Single-handled jar (2003SAY IIM18.5), 6. Single-handled jar with reticulations (2003SAY IIM68.2), 7. Single-handled jar with triangles (2003SAY IIM60.2), 8. Single-handled pot with serrate patterns (2003SAY IM20.5), 9. Single-handled cup with flame-like patterns (2003SAY IIM12.2), 10. Single-handled jar with waves (2003SAYIM89.2), 11. Pottery basin (2003SAY IIIM2.4), 12. Pottery bowl with four legs (2003SAY IM123.1).

A large number of painted pottery vessels were excavated from the Yanghai Tombs. The vessels were made in various shapes with a variety of decorations and include single-handled jars, single-handled cups, four-legged basins, single-handled pots, and various types of bowls. The design elements are rich and display a wide diversity. Such elements include triangles, bands, reticulation, serrate patterns, folded lines, whirlpools, waves, and flame-like patterns (Fig. 3). The colorful decorations may reflect the aesthetics and spiritual views of the ancient Yanghai people.

**Figure 4:**
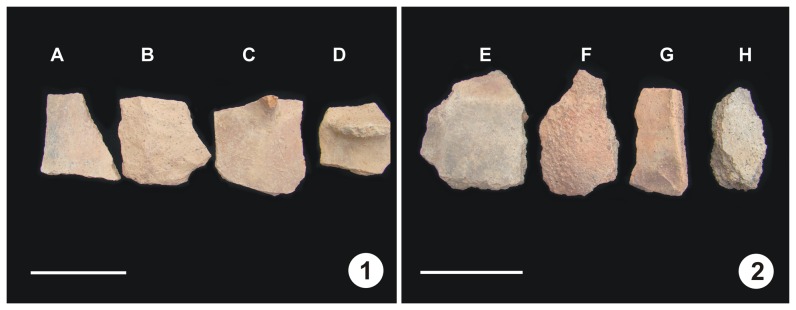
Fired potsherds from the Yanghai Tombs. 1. Fully fired potsherds from Group No. 1 tomb, 2. Incompletely fired potsherds from Group No. 3 tomb. Scale bar = 7 cm.

In the field, more than fifteen potsherds were collected from Tombs No. 1 and 3, among which eight pieces were selected for the present study (Fig. 4). From the color, texture and hardness visible to the naked eye, it appears that potsherds from Tomb No. 1 were fully fired, while those from Tomb No. 3 were incompletely fired. To avoid contamination, the potsherds were thoroughly washed several times with distilled water followed by pure alcohol and were air-dried before crushing.

For pollen analysis, 20 g of each sample were treated following standard palynological techniques [17]. The samples were initially heated with 10% HCl for 10 min to remove carbonates followed by washing with distilled water, and the samples subsequently underwent centrifugation followed by decantation of the supernatant. Next, to remove the humic matter, 10% potassium hydroxide (KOH) was added for 10 min in a hot water bath before heavy liquid flotation. The recovered organic residues were later subjected to acetolysis [18]. Five slides from each sample were prepared for pollen count using polyvinyl alcohol with residues being mounted in Canada balsam and subsequently observed with a Leica DM 2500 light microscope.

For phytolith extraction, 10 g of each sample were processed following the methodology proposed by Pearsall [1] with minor modification, i.e., removal of carbonates and certain oxides with 10% HCl followed by boiling in a mixture of concentrated HCl and HNO_3_ in a water bath until the reaction ceased. Subsequently, the organic matter was removed by boiling in H_2_O_2_ in a water bath. Finally, the phytoliths were separated by heavy liquid flotation (KI+CdI_2_; specific gravity 2.3). For phytoliths, permanent slides were also prepared following the previously described technique for palynomorphs. 100–150 phytoliths were counted and observed in every sample, except for sample B, from which only a few phytoliths were recovered (Table 1).

**Table 1:** Frequency of recovery (total count) of bulliform cell phytoliths from potsherds recovered from Tomb Nos. 1 and 3.

Sample	Dark black/brown colored bulliform cell morphotypes	Transparent bulliform morphotypes
A	53	61
B	21	0
C	51	58
D	66	75
E	12	97
F	14	105
G	17	130
H	15	120

Photographs of palynomorphs and phytoliths were taken under a 40× objective at 400× magnification. For identification, pollen and phytoliths were compared with reference collections from North China and with the published literature and monographs [3]–[5], [11], [19]–[37]. Regarding shape, the recovered phytoliths were categorized following the classification systems proposed by Wang and Lü (1993) [37] and Kondo *et al.* (1994) [27].

## Results

### Pollen analyses

**Figure 5:**
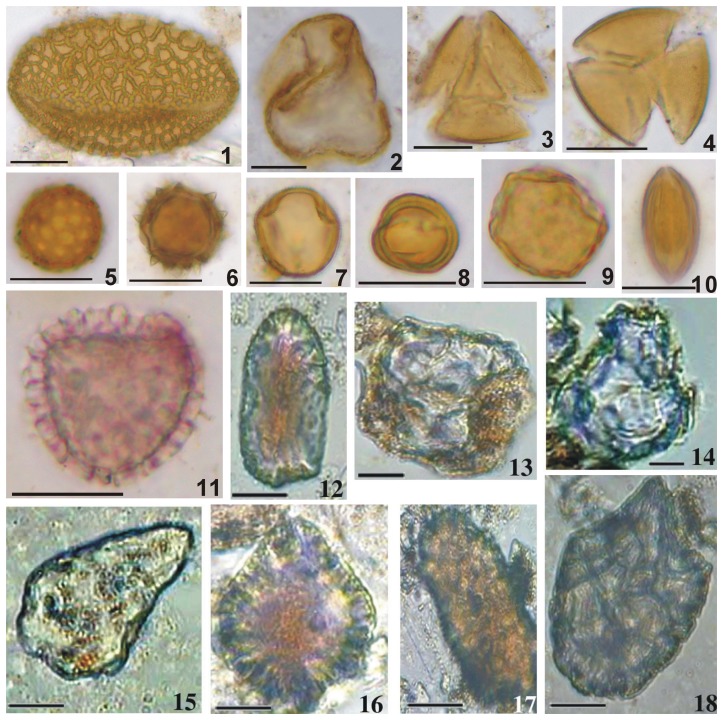
Palynomorphs and phytoliths recovered from ancient potsherds. 1. *Lilium* 2. Deformed Poaceae pollen 3. Gentianaceae 4. Fabaceae 5. Chenopodiaceae 6. Asteraceae 7. *Calophaca* (Fabaceae) 8. *Artemisia* 9. Deformed pollen cf. *Ulmus* 10. *Ephedra* 11. *Lycopodium* 12,17, Dark colored parallepipedal bulliform cell phytoliths 13,14,15. Weathered transparent cuneiform bulliform cell phytoliths. 16,18. Dark colored cuneiform bulliform cell phytoliths Scale bar = 20 µm for Nos. 1–11; Scale bar = 10 µm for Nos. 12–18.

**Figure 6:**
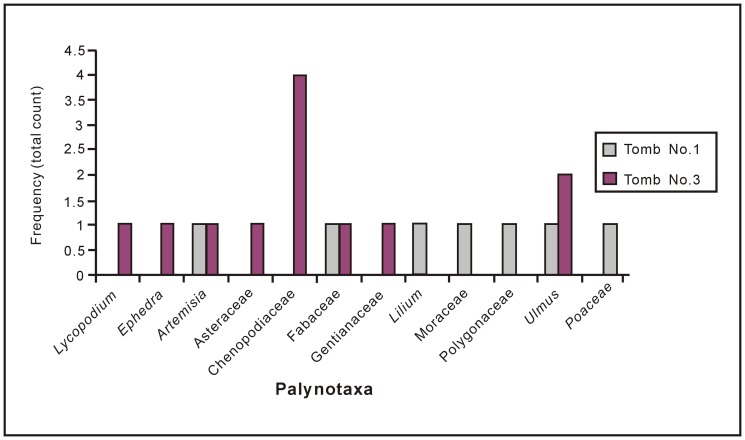
Recovered palynomorphs from potsherds and their frequency distribution.

The fully fired samples from Tomb No. 1 are mostly barren of pollen grains, except for samples A and D, which yielded seven angiosperm palynotaxa (recovery rate: 1.75), one grain each of wormwood (*Artemisia*), lily (*Lilium*), elm (cf. *Ulmus*), buckwheat family (Polygonaceae), legume family (Fabaceae), mulberry family (Moraceae), and grass family (deformed Poaceae pollen). In fully fired potsherds, palynomorphs generally have been destroyed by high temperature during firing. Occasionally, a small number of well-preserved pollen grains (Fig. 5-1) and deformed pollen grains (Fig. 5-2) survived the firing process (Fig. 6).

In all, twelve pollen grains were retrieved from incompletely fired potsherds (samples E, F, G and H) from Tomb No. 3 (recovery rate: 3). The palynoassemblage consists of one pteridophyte taxon (clubmoss; *Lycopodium* sp.), one gymnosperm taxon (ephedra; *Ephedra* sp.), and six angiosperm taxa (e.g., *Artemisia*, cf. *Ulmus*, goosefoot family (Chenopodiaceae), aster family (Asteraceae), Fabaceae, and gentian family (Gentianaceae)) (Fig. 6). The recovery rate of palynomorphs was greater in incompletely fired potsherds than in fully fired samples. Lower temperature during incomplete firing and/or thicker exine of the recovered taxa were probably responsible for the higher recovery rate of identifiable palynomorphs [6], [38].

### Phytolith analyses

**Figure 7:**
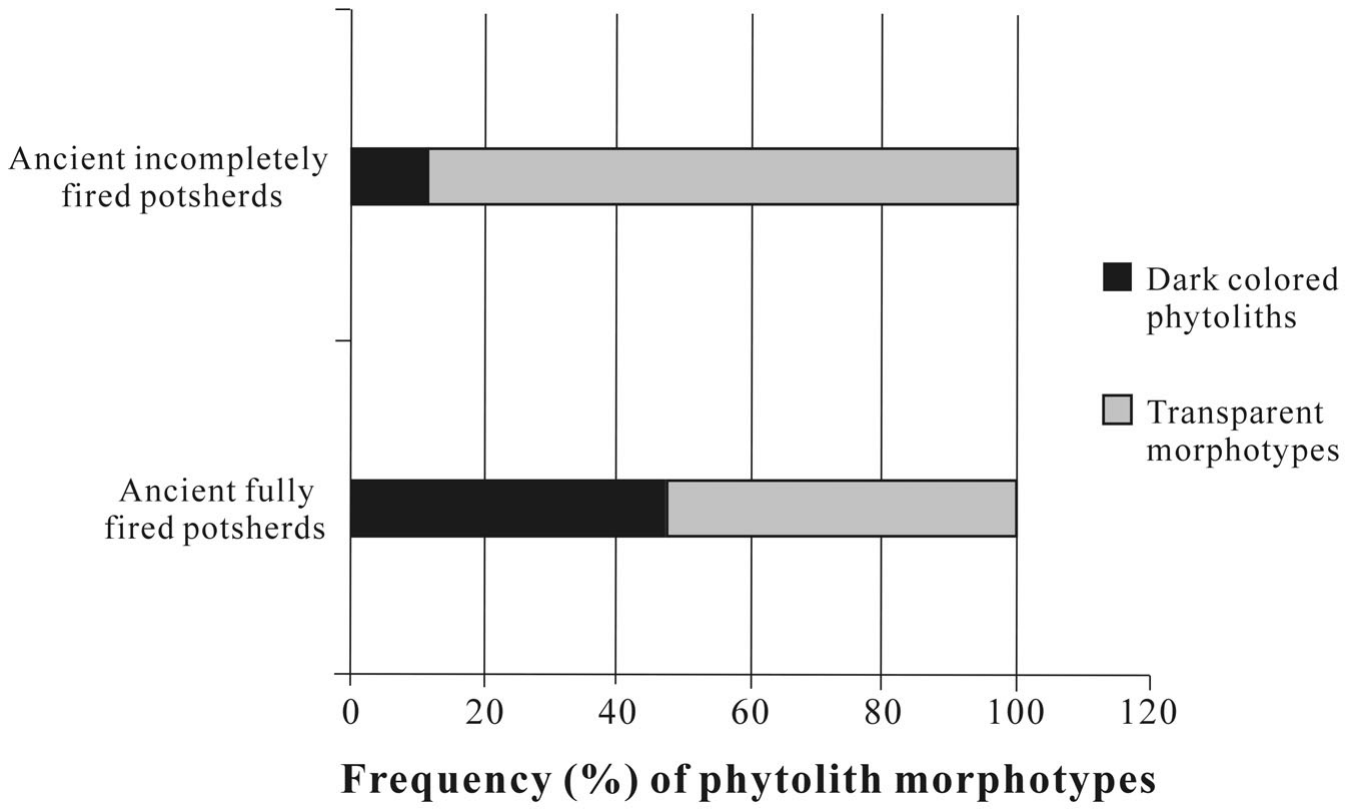
Frequency of phytolith morphotypes in ancient incompletely and fully fired potsherds.

Samples A, C and D of the fully fired potsherds from Tomb No. 1 yielded only abundant fan-shaped or cuneiform and parallepipedal bulliform cell morphotypes of different colors, sizes and shapes (Table 1). However, bulliform cell phytoliths were rare in sample B (Table 1). Bulliform cell morphotypes retrieved from potsherds show differences in color and surface textures, i.e., a portion are transparent, whereas others are dark brown to black at the centers with clearer edges or are sometimes completely black (Figs. 5, 7). The studied potsherds from Tomb No. 1 yielded, on average, 46.7% dark brown to black bulliform cell phytoliths and 53.3% transparent morphotypes (except for sample B). All of the incompletely fired potsherds from Tomb No. 3 (E, F, G and H) also yielded only bulliform cell phytoliths (both cuneiform bulliform cell phytoliths and parallepipedal types). However, the frequency of recovery of transparent bulliform morphotyes is higher (88.6% on average) compared to the dark brown to black colored ones (11.4% on average) (Figs. 5, 7).

Interestingly, most of the recovered large transparent bulliform phytoliths show evidence of weathering with a large number of cavities present on the surface in comparison with the dark colored morphotypes (Figs. 5, 7).

## Discussion

### Frequency analyses of pollen and phytoliths recovered from potsherds

Only a small number of pollen taxa have been retrieved from the potsherds. The frequency of pollen from fully fired potsherds is lower than the frequency of pollen from incompletely fired ones (Fig. 6, total count: 7 vs. 12). Pollen grains with thick and ornamented exines are more resistant to fire-related degradation. Moreover, the nexine shows greater stability at high temperatures than the sexine, and either remains unaltered with some remnants of the sexine, or becomes altered and amorphous [38]. In the present study, all seven pollen taxa that were recovered from fully fired potsherds may have escaped firing at high temperature (i.e., ∼1000°C–1100°C) due to their ornamented thick exine (>2.5–7 µm; Table 2) [38], [39]. In contrast, the rate of recovery of pollen taxa both in number and frequency is enhanced in incompletely fired potsherds, although the small sample size precludes a complete assessment.

**Table 2:** Thickness of exine of palynomorphs recovered from Tomb Nos. 1 and 3.

Palynomorphs	Thickness of exine (µm)
*Lycopodium*	8.5
*Lilium*	7.0
Polygonaceae	6.0
Fabaceae	3.5
*Artemisia*	3.0
Asteraceae	3.0
Chenopodiaceae	3.0
cf. *Ulmus*	2.6
*Ephedra*	2.5
Gentianaceae	2.5
Moraceae	2.5
Poaceae	2.5

The color of bulliform cell phytoliths may serve as a reliable direct indicator of exposure to fire [40]. On the other hand, frequency of occurrence of highly weathered bulliform morphotypes displays a positive relationship with wet and warm climatic conditions [11]. The rate of recovery of charred dark colored bulliform cell phytoliths is higher (76.7% of the total dark colored bulliform cells) in ancient fully fired potsherds than from incompletely fired ones (23.3%) (Table 1). In contrast, the rate of recovery of weathered and transparent bulliform phytoliths is higher in ancient incompletely fired potsherds (69.7%) than from completely fired ones (∼30.0%) (Table 1). Generally, the temperature required for complete firing of an earthenware object ranges from 1000–1100°C in a kiln [39], while incomplete firing at a lower temperature or for a shorter duration results in incompletely fired potsherds. Under the oxidative conditions of firing during the exposure to flame, a persistent change in color of the phytoliths takes place that is retained even after acid treatment [40]. Consequently, higher recovery of dull dark brown/black bulliform phytoliths from fully fired potsherds than from under-fired potsherds indicates that the potsherds recovered from Tomb No. 1 were subjected to a higher temperature and longer duration of firing than those from Tomb No. 3. Moreover, deformations on the surface of a few dark colored bulliform morphotypes in fully fired potsherds might be the result of such elevated temperatures [1], [41].

### Vegetation and climate of Turpan ca. 2700 yrs BP

The palynotaxa found in fully fired and incompletely fired potsherds are listed in Table 2. Previously, a large number of macrobotanical remains recovered from the Yanghai Tombs were reported, including hemp (*Cannabis sativa*) [42], grape (*Vitis vinifera*) [13], common gromwell (*Lithospermum officinale*) [15], caper (*Capparis spinosa*) [43], wolfberry (*Lycium ruthenicum*), foxtail-like sophora (*Sophora alopecuroides*), small aeluropus (*Aeluropus pungens* var. *pungens*), charab poplar (*Populus euphratica*), showy chloris (*Chloris virgata*), common reed (*Phragmites australis*), barnyard grass (*Echinochloa crus-galli*), willow (*Salix* sp.), spruce (*Picea* sp.) [44], and some cereal remains, including broomcorn millet (*Panicum miliaceum*), hull-less barley (*Hordeum vulgare* var. *nudum*), and bread wheat (*Triticum aestivum*) [45].

Based on the climatic preferences of the above palynotaxa and plant remains, it is possible to make several suppositions about the vegetation and climate of Turpan ca. 2700 yrs BP. For instance, *Capparis spinosa* has a natural distribution in the temperate zone of the Northern Hemisphere of the Old World from southern Europe, northern and eastern Africa, Madagascar, southwestern and central Asia to Australia and Oceania [46], [47]. *Salix* species are mostly distributed in the cold and temperate regions of the Northern Hemisphere, with a few being observed in the Southern Hemisphere. *Artemisia* usually grows in dry areas of the North Temperate Zone, mainly in Europe and China. *Ulmus* is commonly distributed in the North Temperate Zone of Asia, Europe, and North America. *Lilium* mostly grows in the temperate and alpine regions of the Northern Hemisphere, especially in East Asia [48]. It is assumed, therefore, that temperate vegetation and a correspondingly arid climate existed in Turpan ca. 2700 yrs BP. We also envision the landscape at 2700 yrs BP as follows: there was a swamp existing in the oasis where *Phragmites*, Polygonaceae and several species of ferns were growing. Trees, e.g., *Populus euphratica* and *Salix* sp., grew on the edges of the swamp. On the periphery of the oasis, there was grassland with *Artemisia*, *Ephedra*, Chenopodiaceae and Asteraceae. On the nearby mountains, certain conifers (e.g., *Picea*) were growing. The ancient Yanghai people settled down in the oasis and started planting cereal crops (e.g., broomcorn millet, hull-less barley and bread wheat), but they also led a life of hunting and pastoralism as evidenced by saddlery, bows, arrows, and wool unearthed from the Yanghai Tombs [45], [49].

Bulliform cell morphotypes retrieved from both fully fired potsherds and under-fired ones from Tomb No. 1 and Tomb No. 3 might have been contributed by *Phragmites* inhabiting the swamp in the oasis and by the surrounding peripheral grass cover, which enrich the palynological and macrobotanical data. Because highly weathered bulliform phytoliths are directly correlated to warm and wet climatic conditions [11], the occurrence of higher frequencies of weathered transparent bulliform morphotypes in both fully fired and under-fired potsherds suggests that warm and wet conditions prevailed in the area. Such wet conditions in an arid region can be explained by the presence of a swamp around the study site that served as a water source, as suggested by the palynological study.
